# Ketone Supplementation Dampens Subjective and Objective Responses to Alcohol: Evidence From a Preclinical Rat Study and a Randomized, Cross-Over Trial in Healthy Volunteers

**DOI:** 10.1093/ijnp/pyae009

**Published:** 2024-02-05

**Authors:** Xinyi Li, Zhenhao Shi, Dustin R Todaro, Timothy Pond, Juliana I Byanyima, Sianneh A Vesslee, Rishika Reddy, Ravi Prakash Reddy Nanga, Gabriel Kass, Vijay Ramchandani, Henry R Kranzler, Janaina C M Vendruscolo, Leandro F Vendruscolo, Corinde E Wiers

**Affiliations:** Center for Studies of Addiction, Department of Psychiatry, University of Pennsylvania Perelman School of Medicine, Philadelphia, Pennsylvania, USA; Center for Studies of Addiction, Department of Psychiatry, University of Pennsylvania Perelman School of Medicine, Philadelphia, Pennsylvania, USA; Center for Studies of Addiction, Department of Psychiatry, University of Pennsylvania Perelman School of Medicine, Philadelphia, Pennsylvania, USA; Center for Studies of Addiction, Department of Psychiatry, University of Pennsylvania Perelman School of Medicine, Philadelphia, Pennsylvania, USA; Center for Studies of Addiction, Department of Psychiatry, University of Pennsylvania Perelman School of Medicine, Philadelphia, Pennsylvania, USA; Center for Studies of Addiction, Department of Psychiatry, University of Pennsylvania Perelman School of Medicine, Philadelphia, Pennsylvania, USA; Center for Studies of Addiction, Department of Psychiatry, University of Pennsylvania Perelman School of Medicine, Philadelphia, Pennsylvania, USA; University of Pennsylvania Perelman School of Medicine, Department of Radiology, Philadelphia, Pennsylvania, USA; Center for Studies of Addiction, Department of Psychiatry, University of Pennsylvania Perelman School of Medicine, Philadelphia, Pennsylvania, USA; National Institute on Alcohol Abuse and Alcoholism, National Institutes of Health, Bethesda, Maryland, USA; Center for Studies of Addiction, Department of Psychiatry, University of Pennsylvania Perelman School of Medicine, Philadelphia, Pennsylvania, USA; National Institute on Drug Abuse, National Institutes of Health, Baltimore, Maryland, USA; National Institute on Drug Abuse, National Institutes of Health, Baltimore, Maryland, USA; Center for Studies of Addiction, Department of Psychiatry, University of Pennsylvania Perelman School of Medicine, Philadelphia, Pennsylvania, USA

**Keywords:** Ketone supplements, alcohol use disorder, alcohol intoxication, alcohol sensitivity, nutritional ketosis

## Abstract

**Background:**

Previous preclinical and human studies have shown that a high-fat ketogenic diet and ketone supplements (KS) are efficacious in reducing alcohol craving, alcohol consumption, and signs of alcohol withdrawal. However, the effects of KS on alcohol sensitivity are unknown.

**Methods:**

In this single-blind, cross-over study, 10 healthy participants (3 females) were administered a single, oral dose of a KS (25 g of ketones from D-β-hydroxybutyric acid and R-1,3-butanediol) or placebo 30 minutes before an oral alcohol dose (0.25 g/kg for women; 0.31 g/kg for men). Assessments of breath alcohol concentration and blood alcohol levels (BAL) and responses on the Drug Effect Questionnaire were repeatedly obtained over 180 minutes after alcohol consumption. In a parallel preclinical study, 8 Wistar rats (4 females) received an oral gavage of KS (0.42 g ketones/kg), water, or the sweetener allulose (0.58 g/kg) followed 15 minutes later by an oral alcohol dose (0.8 g/kg). BAL was monitored for 240 minutes after alcohol exposure.

**Results:**

In humans, the intake of KS before alcohol significantly blunted breath alcohol concentration and BAL, reduced ratings of alcohol liking and wanting more, and increased disliking for alcohol. In rats, KS reduced BAL more than either allulose or water.

**Conclusion:**

KS altered physiological and subjective responses to alcohol in both humans and rats, and the effects were likely not mediated by the sweetener allulose present in the KS drink. Therefore, KS could potentially reduce the intoxicating effects of alcohol.

**Significance Statement**

Previous research has suggested the ketogenic diet and ketone supplements (KS) as potential therapies for alcohol use disorder, but to our knowledge, no studies have examined the effects of KS on alcohol sensitivity. Here, we demonstrated that pairing alcohol with KS dampened breath alcohol concentration and blood alcohol levels, reduced ratings of alcohol liking and wanting, and increased disliking for alcohol in healthy human participants. A parallel rat study replicated the finding and additionally demonstrated that the low-calorie sweetener allulose in the KS drink did not contribute to the blunted blood alcohol responses. Therefore, KS may have clinical relevance for reducing the intoxicating effects of alcohol and, consequently, alcohol-related harm.

## INTRODUCTION

The 2021 National Survey on Drug Use and Health estimated that approximately 21.5% of US individuals aged 12 years or older binge drank alcohol in the past month and that 10.2% of individuals met DSM-5 diagnostic criteria for alcohol use disorder (AUD) (Substance Abuse and Mental Health Services Administration, 2022). Excessive alcohol consumption increases the risk for reckless behaviors (e.g., violence and driving under the influence) and pathological conditions, including cognitive decline, liver disease, and certain types of cancer, and is one of the leading causes of preventable deaths in the United States (Esser et al., 2022).

Nutritional ketosis has emerged as a potential therapy for the treatment of alcohol withdrawal and AUD (Mahajan et al., 2021; Wiers et al., 2021). Nutritional ketosis is a metabolic state characterized by elevated levels of ketone bodies, which collectively refer to acetone, acetoacetate, and β-hydroxybutyrate (BHB). Ketosis can be achieved through fasting or adherence to a high-fat, low-carbohydrate ketogenic diet. Preclinical studies have shown the efficacy of a ketogenic diet in reducing alcohol consumption (Blanco-Gandía et al., 2021; Wiers et al., 2021) and alcohol withdrawal (Dencker et al., 2018; Bornebusch et al., 2021). These findings were corroborated by human studies demonstrating that a ketogenic dietary intervention attenuated alcohol craving and wanting (Castro et al., 2018; Wiers et al., 2021) and the need for benzodiazepine medication for alcohol withdrawal management in AUD inpatients (Wiers et al., 2021). However, given the restrictive nature of the ketogenic diet, adherence has proven difficult. Exogenous ketone supplements (KS) offer an alternative and have been shown to elevate serum levels of ketone bodies without dietary deprivation of carbohydrates (Stubbs et al., 2017). Preclinical studies have shown KS to reduce alcohol withdrawal symptoms and reinforcement similarly to a ketogenic diet (Bornebusch et al., 2021). However, the mechanisms underlying the efficacy of ketosis in reducing alcohol withdrawal, consumption, and craving or wanting are poorly understood and may involve physiological interactions with alcohol.

Alcohol is absorbed in the gastrointestinal tract, from which it enters the circulation for distribution throughout the body (Zakhari, 2006; Cederbaum, 2012). Alcohol is metabolized in the liver through sequential oxidation to acetaldehyde and acetate by alcohol and aldehyde dehydrogenases (ADH), respectively. It is also metabolized to a lesser extent in nonhepatic organs, such as the stomach and the brain (Zakhari, 2006). A preclinical study demonstrated 5-fold higher blood alcohol levels (BAL) following alcohol vapor exposure in rats that were maintained on a 6-week ketogenic diet compared with a standard chow diet (Wiers et al., 2021). These findings may be explained in part by the presence of ketones and/or the restriction of carbohydrates with a ketogenic diet because both carbohydrate-rich diets (Finnigan et al., 1998) and sugar consumption (Stamates et al., 2015; Smith et al., 2017) reduce breath alcohol concentration (BrAC) and BAL in responses to oral alcohol challenges. In contrast, oral exogenous KS in the presence of a regular, carbohydrate-rich diet significantly reduced BAL in mice compared to those maintained on a regular diet without KS or a ketogenic diet (Bornebusch et al., 2021).

Here, we compared the acute effects of a KS with placebo on BrAC and BAL responses to an alcohol challenge in healthy human volunteers. We also assessed subjective responses to alcohol after KS and placebo on the Drug Effect Questionnaire (DEQ), which includes questions regarding feelings of intoxication, alcohol liking, and wanting. Because the low-caloric sweetener allulose was present in our KS drink, we also compared the effects of KS with those of allulose on BAL responses to an oral alcohol challenge in rats. We hypothesized that, in contrast to the ketogenic diet study reporting elevated BAL (Wiers et al., 2021) but consistent with the previous findings on KS reducing BAL (Bornebusch et al., 2021), KS would reduce both objective (BrAC and BAL in both humans and rats) and subjective responses (in humans) to a moderate alcohol challenge.

## MATERIALS AND METHODS

### Human Study

#### Participants

Twelve healthy individuals aged 21 to 50 years were recruited for participation in the study. Participants self-reported to have consumed at least 2 standard alcohol drinks on a single day at least once in the prior month. Two individuals withdrew from the study, and n = 10 participants (3 females and 7 males) completed both study arms. Participants' characteristics are provided in Table 1. Participant recruitment started in October 2022, and the study concluded in June 2023, after 10 participants completed the study (see supplementary Figure 1 for CONSORT Diagram). The study was performed at the University of Pennsylvania, approved by the Institutional Review Board at the University of Pennsylvania, and registered at ClinicalTrials.gov (NCT05551754).

**Table 1.** Participants’ characteristics (mean ± SD)

Characteristics	
Age	29.7 ± 6.8
Sex at birth	3 females/7 males
BMI (kg/m^2^)	25.3 ± 4.6
Race	1 African American/1 Asian/8 White
Highest education completed	2 some college/6 college/2 advanced degrees
Standard alcohol drinks/week^a^	3.5 ± 2.3

Abbreviation: BMI, body mass index.

^a^Assessed using 90-day timeline follow-back at screening.

Participants were screened and excluded if they had any major medical conditions (e.g., gastrointestinal disorders, including irritable bowel syndrome, liver disease, kidney disease, or metabolic disorders, including diabetes) or were taking medication that could interfere with study procedures (e.g., the use of KS and alcohol). Individuals with a major psychiatric or substance use disorder (other than nicotine or cannabis use disorder) based on medical history and the structured Mini-International Neuropsychiatric interview for the Diagnostic and Statistical Manual of Mental Disorders, 5th ed (American Psychiatric Association, 2013) were excluded from participation. Also excluded from participation were females who were pregnant or lactating, individuals weighing more than 102 kilograms (to limit the volume of alcohol provided to individuals), individuals positive for any substance other than cannabis on a urine drug test, or those who had a baseline breath alcohol level above 0.00% on the day of the study procedures.

#### Study Design

**Figure 1.**
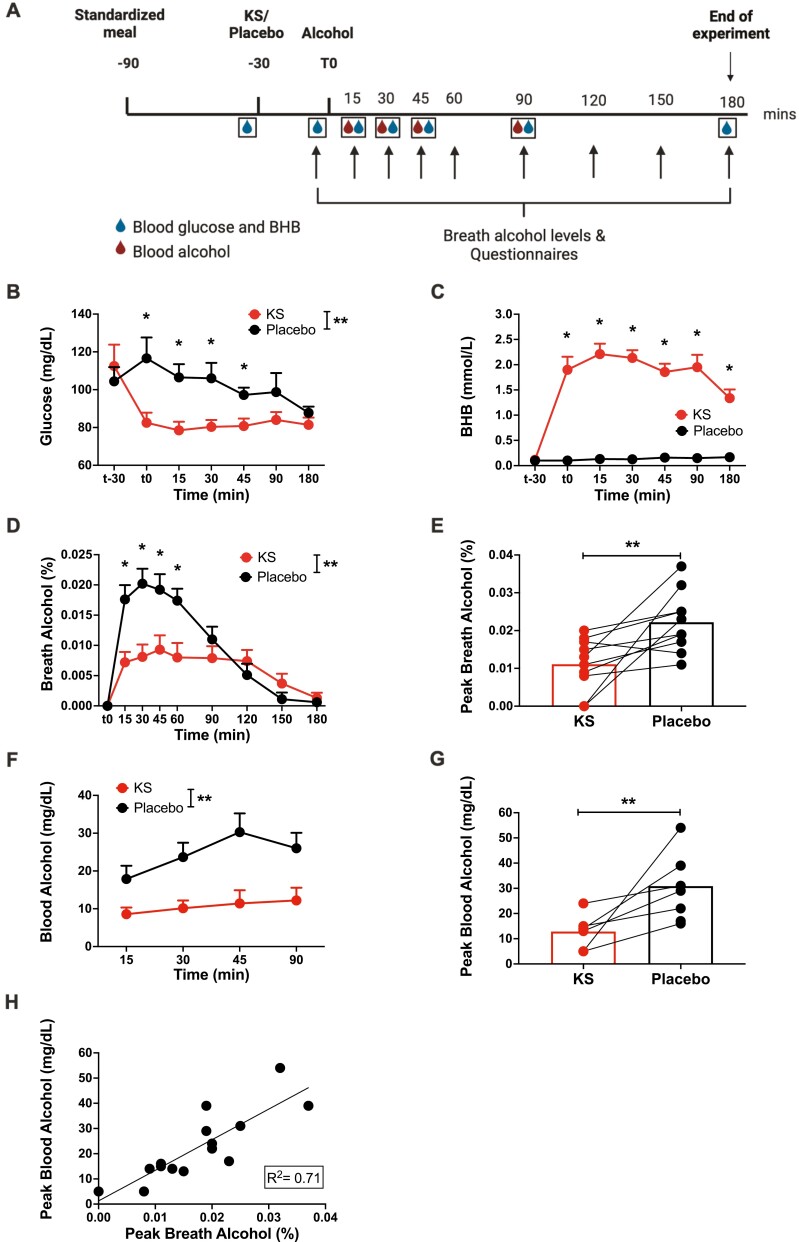
Responses following an alcohol challenge paradigm in individuals administered ketone supplements (KS) and placebo. (A) Schematic of the experimental timeline (human). Participants arrived at the study center following an overnight fast. They received a standardized meal, the KS or placebo approximately 1 hour later, and then an oral dose of alcohol 30 minutes later. Breath alcohol concentration (BrAC, 9 times, arrows), blood alcohol level (BAL, 4 times, red drops), blood β-hydroxybutyrate (BHB) and glucose levels (7 times, blue drops), and subjective ratings (5 times) were assessed. (B) Measures of blood glucose at t-30 (pre-intervention) (*n* = 5/5; KS/placebo), t0 (pre-alcohol) (*n* = 6/5), and 15 (*n* = 10/10), 30 (*n* = 6/4), 45 (*n* = 10/10), 90 (*n* = 6/4), and 180 (*n* = 10/9) minutes following alcohol administration. (C) Measures of blood BHB at t-30 (pre-intervention) (*n* = 6/5; KS/placebo), t0 (pre-alcohol) (*n* = 6/6), and 15 (*n* = 10/10), 30 (*n* = 6/4), 45 (*n* = 10/10), 90 (*n* = 6/4), and 180 (*n* = 9/8) minutes following alcohol administration. (D) Measures of BrAC before (t0) (n = 10/10; KS/placebo) and at 15, 30, 45, 60, 90, 120, 150, and 180 minutes (n = 10/10) following alcohol administration. (E) Comparison of peak BrAC between placebo and KS. (C) Measures of BAL at 15 (*n* = 8/7; placebo/KS), 30 (*n* = 6/7), 45 (*n* = 7/5), and 90 (*n* = 6/5) minutes following alcohol administration. (D) Comparison of peak BAL between KS and placebo. (E) Correlation between peak BAL and BrAC. Mean ±SEM. **Indicates significant main intervention effect. *Indicates significant differences between placebo and KS at a particular time point, *P *< .05. Abbreviations: BHB, β-hydroxybutyrate; KS, ketone supplement.

In this single-blinded, cross-over study, participants received KS and placebo on 2 separate study visits with a minimum 1-day washout period in between. The order in which participants received the KS and placebo was randomized and counterbalanced. Participants were asked to arrive following an overnight fast and were provided a standardized meal (of approximately 550 kcal). Approximately 1 hour later, participants drank the KS or placebo, followed by an oral dose of alcohol 30 minutes later (Figure 1A). All adverse events were mild and transient, and participants reported resolution of the adverse effects during follow-up phone calls the day after the study visit. A list of adverse events is provided in supplementary**Adverse Events** and supplementaryTable 1.

#### KS and Placebo

The KS and placebo were supplied by Vitanav Inc, Washington DC, USA. The KS (147.5 mL, Kenetik, Ketone Concentrate) contained 25 g ketones from a mixture of D-β-hydroxybutyric acid and R-1,3-butanediol and 35 g of the low-calorie sweetener allulose (total 150 kcal). The placebo contained 74 mL Safeway Sweet and Sour mix, 18.5 mL lemon juice, 18.5 mL lime juice, and 37 mL water (total 57.2 kcal from 14.3 g of sugar) (Vitanav Inc). The study drinks were diluted with water on the day of the study visit. The order in which the participants received the KS and placebo drinks was randomized, and participants were blinded to the study intervention. After study participation, participants were asked which intervention they thought they received and rated their confidence in their guesses (0-10 scale).

#### Alcohol Challenge

The dose of alcohol (100 proof Smirnoff vodka, Diageo plc, London, UK) was adjusted for body weight and sex to achieve a target breath alcohol level of 0.05% (0.25 g/kg for women and 0.31 g/kg for men). The alcohol was diluted in sugar-free soda according to the participants’ preference, and the participants were asked to finish the alcoholic drink within 5 minutes. Objective and subjective measures of alcohol intoxication were monitored before and for 180 minutes following alcohol intake. BrAC were assessed by handheld breath alcohol monitors (Intoximeters Inc., St. Louis, MO, USA). Blood was collected by trained study staff and sent to the laboratory at the Hospital of the University of Pennsylvania for BAL analyses. Due to difficulties in blood draws, several BALs were missed (Ns for each time points are presented in the figure legends).

Self-reported assessments included the DEQ (Preston and Bigelow, 1991) consisting of 5 items: (1) FEEL the effects of alcohol, (2) feel HIGH, (3) DISLIKE any of the effect you are feeling, (4) LIKE the effect you are feeling, (5) want MORE of what you consumed right now, which were rated on a 100-mm visual analog scale that ranges from not at all (0) to a lot (100). The Brief Biphasic Alcohol Effect Scale (BAES) (Martin et al., 1993) assessed whether participants were energized, excited, sedated, sluggish, up, or had slow thoughts, on a 10-point scale from not at all (0) to extremely (10). The responses were combined in the 2 sum scores: stimulation (energized, excited, and up) and sedation (sedated, slow thoughts, and sluggish). The 8-item Alcohol Urge Questionnaire (AUQ) (Bohn et al., 1995) assessed alcohol craving. Responses on the DEQ and BAES were missing in 1 placebo participant at 1 and 2 time points, respectively.

Blood levels of β-hydroxybutyrate (BHB) and glucose were collected before the KS/placebo intervention and monitored throughout the study using finger pricks and commercially available monitors (Precision Xtra, Abbott Laboratories, Chicago, IL, USA) (Ns for each time points are presented in the figure legends). Participants were provided a snack at the end of the study. In cases during which participants had blood glucose levels below 70 mg/dL (as was the case with 2 participants following the KS intervention trial), snacks were provided upon measurement of the blood sugar, which was continously monitored until it reached more than 70 mg/dL. Participants were called the next business day to inquire of any possible adverse events that they may have experienced.

#### Data Analyses

We performed power analysis using G*Power 3.1 with a focus on the F-test of repeated measures of the difference in the primary BrAC outcome variable between KS and placebo. Based on an estimate of a correlation of 0.3 among repeated measures, a sample of 10 individuals would provide a statistical power of β > .80 to detect a large effect of Cohen’s f = 0.40 at the threshold of α = .05.

All analyses were conducted with SPSS (IBM Corp., Armonk, NY, USA) using linear mixed-effects analyses to examine the main effect of intervention (KS vs placebo), the main effect of time (repeated assessments over time), and the time × intervention interaction effect. We included visit order (KS/placebo or placebo/KS) as a covariate and individual-specific random intercepts in the model. The study’s primary outcome measures were BrAC and BAL responses to alcohol following KS vs placebo. For BAL, measurements below the laboratory detection cutoff of 10 mg/dL were analyzed as 5 mg/dL, as per Farokhnia et al. (Farokhnia et al., 2021). Secondary outcome measures were the effects of KS vs placebo on the DEQ. Subjective responses to KS/placebo before alcohol intake were measuredand compared using paired Student *t-*tests. To correct for the potentially confounding effects of these pre-alcohol DEQ responses, we reported the linear mixed-effects analyses on DEQ responses both uncorrected and corrected for pre-alcohol ratings. When applicable, post hoc pairwise comparisons were analyzed with Bonferroni correction.

In exploratory analyses, we used linear regression to examine the congruency between peak BAL and BrAC during the KS and placebo intervention arms, and linear mixed-effects analyses to examine the effects of the KS/placebo intervention on blood glucose and BHB levels, BAES, and AUQ. Moreover, paired Student *t-*tests were used to compare peak BrAC and BAL following KS and placebo.

### Rat Study

A total of 8 Wistar rats (4 males and 4 females) with ad libitum access to water and food were group-housed in a temperature- and humidity-controlled facility and maintained on a reverse 12-hour-light/-dark cycle (lights on at 6:00 am). All procedures were conducted according to the National Institutes of Health Guide for the Care and Use of Laboratory Animals and were approved by the National Institute on Drug Abuse, Intramural Research Program, Animal Care and Use Committee.

The rats received a dose of Kenetik (0.42 g ketones/kg), allulose (0.58 g/kg; Wholesome Sweeteners Inc., Sugar Land, TX, USA), or water at a volume of 2.45 mL/kg body weight through oral gavage. After 30 minutes, 0.8 g/kg alcohol was administered orally. Blood was collected from the tip of the tail before and at prespecified time intervals following alcohol administration (Figure 3A). Blood glucose (glucometer, Tyson Bioresearch Inc., Zhunan, Taiwan) and BHB (Precision Xtra Blood Ketone Monitoring System, Abbott, Alameda, CA, USA), and alcohol levels were measured. BALs were assessed using gas chromatography-mass spectrometry, in which an ethanol calibration curve was prepared from 12.5 to 300 mg/dL using ethanol standards in water (Cerilliant, Round Rock, TX, USA). The 50-mg/dL calibrator was diluted with water to achieve calibrators at the 12.5-and 25-mg/dL levels. Briefly, 10 µL ethanol standard or whole blood was added to 10-mL glass headspace vials (Agilent, Santa Clara, CA, USA) and sealed with a crimp cap. The vials were heated in a 70°C 7697A Headspace Sampler (Agilent) before headspace injection onto an MXT-Volatiles 30-m, 0.28-mm ID, 1.25-µm df column (Restek, Centre County, PA, USA) using helium as the carrier gas. The 8890 GC System column oven (Agilent) was heated to 40°C for an isocratic 6-minute run paired with a 5977B GC/MSD (Agilent).

**Figure 2.**
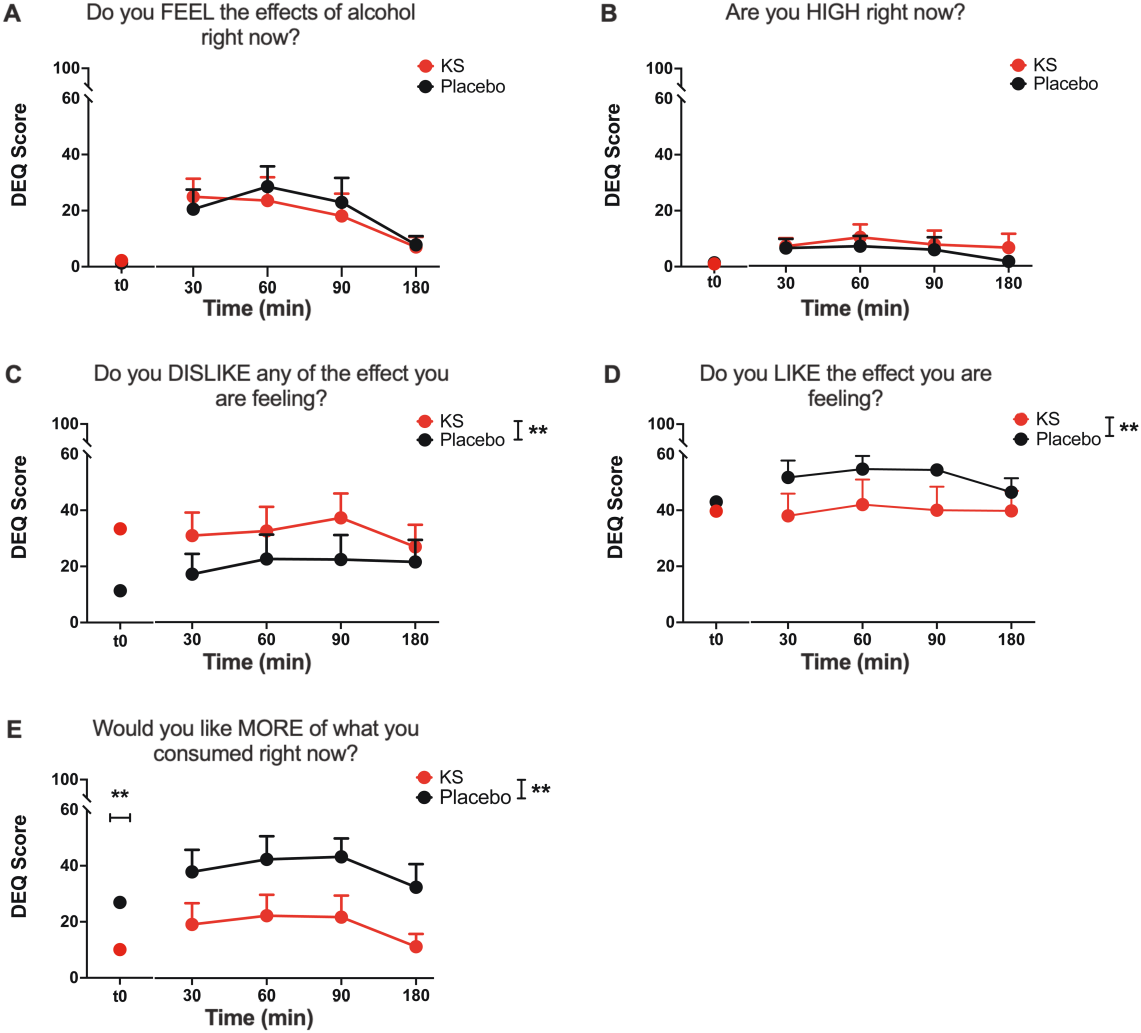
Drug Effect Questionnaire responses before and following an alcohol challenge paradigm in individuals administered KS or placebo. (A) Subjective responses regarding (A) FEEL the effects of alcohol, (B) reports of HIGH, (C) DISLIKE the effects, (D) LIKE the effects, (E) like MORE of what you consumed assessed before and at 30, 60, 90, and 180 minutes following the alcohol challenge. Mean ±SEM. **Indicates significant main effect of intervention, unadjusted, *P *< .05. Abbreviations: DEQ, Drug Effect Questionnaire; KS, ketone supplement.

**Figure 3.**
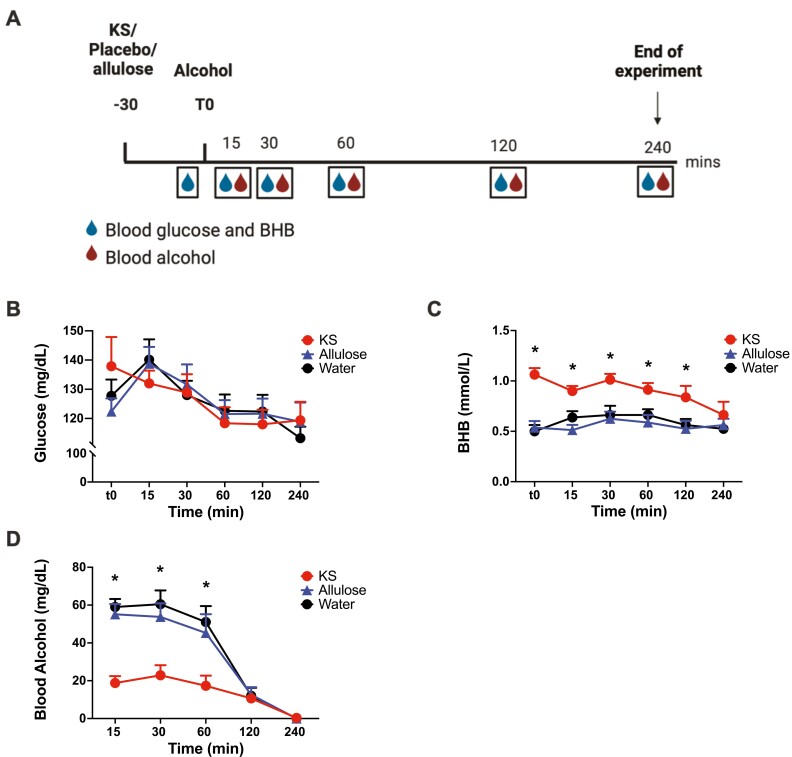
Responses levels following an oral alcohol challenge in rats administered ketone supplements (KS), allulose, or water. (A) Experimental timeline. Rats were administered KS, allulose, or water by oral gavage 30 minutes before an oral gavage dose of alcohol. Blood was collected for assessments of blood alcohol levels (BAL, 5 times, red drops) and blood β-hydroxybutyrate (BHB) and glucose levels (6 times, also collected at 0 minutes; blue drops). T0 corresponds to the time of alcohol administration. (B) Blood glucose and (C) BHB levels were assessed immediately before (t0, 30 minutes following KS, allulose, or water administration) and at 15, 30, 60, 120, and 240 minutes after alcohol challenge. (D) Assessments for BAL were made at 15, 30, 60, 120, and 240 minutes after oral administration of alcohol. Data are expressed as mean ±SEM. *Indicates significant KS differences from water and allulose at a particular time point, *P *< .05. BHB, β-hydroxybutyrate; KS, ketone supplement

Blood glucose, BHB, and alcohol levels were analyzed using linear mixed-effects models with individual-specific random intercepts to test for the main effects of ketone treatment, sex, and time, as well as their interactions. If applicable, post hoc pairwise comparisons were conducted with Bonferroni correction.

## RESULTS

### Human Study

#### Blood Glucose and BHB

For blood glucose levels, there were significant effects of intervention (F_1,78.0_=27.6, *P *< .001), time (F_6,77.9_ = 5.7, *P *< .001), and the intervention × time interaction (F_6,77.9_ = 5.7, *P* = .008). Post hoc analyses showed significantly lower blood glucose levels for up to 75 minutes following KS vs placebo administration (Bonferroni corrected, *P *< .05) (Figure 1B).

For blood BHB, there were significant effects of intervention (F_1, 76.0_ = 418.2, *P *< .001), time (F_6,75.4_ = 11.3, *P *< .001), and the intervention × time interaction (F_6,75.4_ = 11.3, *P *< .001). Post hoc analyses demonstrated significantly higher blood BHB levels following KS, which peaked at 45 minutes after KS intake and remained elevated throughout the 180-minute alcohol challenge study, compared with placebo (Bonferroni corrected, *P *< .05) (Figure 1C).

#### Breath and Blood Alcohol Concentrations

For BrAC, there were significant effects of intervention (F_1,152.0_ = 39.0, *P *< .001), time (F_8,152.0_ = 28.5, *P *< .001), and the intervention × time interaction (F_8,152_ = 7.7, *P *< .001) (Figure 1d). Post hoc analyses indicated significantly lower BrAC following KS than placebo for up to 60 minutes after alcohol consumption (Bonferroni corrected, *P *< .05). Furthermore, paired Student *t*-test demonstrated significantly lower peak BrAC with KS than placebo (t_9_ = 3.2, *P* = .010) (Figure 1E). BrAC data for individual participants following KS and placebo are presented in supplementary Figure 2.

For BAL, there was a significant effect of intervention (F_1,37.3_ = 27.9, *P *< .001), with lower BAL with KS. However, there were no effects of time (F_3,37.0_ = 1.9, *P* = .2) or the intervention × time interaction (F_3,37.3_ = 0.8, *P* = .5) (Figure 1F). Lower peak BAL was observed following the KS intervention than placebo (t_6_ = 3.6, *P* = .012) (Figure 1G). Exploratory linear regression with both KS and placebo measures pooled together demonstrated a significant association between peak BrAC and BAL measures (F_1,14_ = 232.5, *P *< .001, R^2^ = 0.71) (Figure 1H).

#### Subjective Ratings: DEQ

No effects of intervention were observed for DEQ ratings of FEEL (Figure 2a) or HIGH (Figure 2B) at t0 (i.e., 15 minutes after KS/placebo but before alcohol administration) or following alcohol intake, but there was a significant main effect of time on FEEL the effects of alcohol (F_3,60.2_ = 5.8, *P* = .002)

At t0, participants reported higher DISLIKE at a trend level (t_8_ = −2.17, *P* = .06), no change in LIKE, and lower wanting MORE (t_9_ = 3.0, *P* = .015) for KS vs placebo. After alcohol intake, there were significant main effects of the KS intervention on DEQ ratings of DISLIKE (F_1,57.1_ = 9.4, *P* = .003), LIKE (F_1,56.6_ = 6.8, *P* = .012), and liking MORE (F_1,60._3 = 29.8, *P *< .001), with KS resulting in higher DISLIKE and lower LIKE and wanting MORE ratings of alcohol, but no main effects of time (Figure 2C–E). There was no significant intervention × time interaction (*P*s > .05). The significant main effects of KS intervention remained after correction for pre-alcohol ratings for DISLIKE (F_1,58.8_ = 8.8, *P* = .004) and LIKE (F_1,53.4_ = 5.7, *P* = .02) but not for wanting MORE alcohol (F_1,65.6_ = 0.0, *P* = .9) (see supplementary Analyses of Baseline-Adjusted DEQ Scores for analyses that correct for pre-alcohol ratings).

#### Subjective Ratings: BAES and AUQ

There were no significant effects of intervention, time, or the intervention × time interaction on BAES or AUQ ratings (all *P*s > .05, see supplementary Analyses of BAES and AUQ and supplementary Figure 3).

#### Study Blinding

Eight of 10 participants during the KS and 9 of 10 participants during the placebo visit correctly guessed the study intervention (χ_1_ = 9.9, *P* = .002). Confidence ratings did not differ between the KS (mean = 5.6 ± 3.1) and placebo (5.8 ± 3.0) visits (t_9_ = 0.3, *P* = .8).

### Rat Study

For blood glucose levels, there were significant effects of time (F_5, 90_ = 8.4, *P *< .001) and sex (F_1, 18_ = 45.2, *P *< .001), but no main effect of ketone treatment (F_2, 18_ = 0.0, *P* = 1.0) or any interaction effects (*P* > .05) (Figure 3B; supplementary Figure 5A). For blood BHB levels, there were significant effects of ketone treatment (F_2, 18_ = 17.7, *P *< .001), time (F_5, 90_ = 4.0, *P* = .002), and the ketone treatment × time interaction (F_10, 90_ = 2.4, *P* = .014). Post hoc comparisons indicated that blood BHB was significantly higher up to 150 minutes after KS intake than water and allulose (Bonferroni corrected, *P *< .05) (Figure 3C; supplementary Figure 5B). There were also significant effects of sex (F_1, 18_ = 7.5, *P* = .014) and the time × sex interaction (F_5, 90_ = 2.4, *P* = .046), such that males had higher BHB levels than females at 0, 15, 30, 60, and 120 minutes following alcohol administration (Bonferroni corrected, *P *< .05). No significant effects of the intervention × sex (F_2,18_ = 0.3, *P* = .7) or the ketone treatment × sex ×time (F_10,90_ = 0.8, *P* = .6) interactions were observed.

Analyses of BAL in rats indicated a significant effect of ketone treatment (F_2,20.4_ = 21.9, *P *< .001), time (F_4,70.9_ = 119.2, *P *< .001), sex (F_1,16.2_ = 14.1, *P* = .002), the ketone treatment × time interaction (F_8, 70.0_ = 11.0, *P *< .001), and the sex × time interaction (F_4,70.9_ = 8.6, *P *< .001) but not the ketone treatment × sex interaction (F_2, 20.4_ = 1.9, *P* = .2) or ketone treatment × time × sex interaction (F_8, 70.9_  = 0.6, *P* = .8) (Figure 3D; supplementary Figure 5C). Post hoc pairwise comparisons did not show significant differences in BAL between water and allulose. KS significantly reduced BALs compared with water and allulose up to 15, 30, and 60 minutes following alcohol administration (Bonferroni corrected, *P *< .05). Male rats had higher BAL than females at 30, 60, and 120 minutes following alcohol administration (Bonferroni corrected, *P *< .05) (supplementary Figure 5C).

## DISCUSSION

Emerging studies have shown a role for nutritional ketosis, induced through a ketogenic diet or exogenous KS, in alleviating symptoms of alcohol withdrawal and alcohol craving (Bornebusch et al., 2021; Mahajan et al., 2021; Wiers et al., 2021). Here, we found evidence in both humans and rats that the administration of a KS before alcohol significantly attenuated BAC and BALs. In humans, KS additionally reduced subjective ratings of alcohol liking and wanting and increased alcohol disliking. However, no differences in reports of feeling high, feeling the effects of alcohol, or feeling stimulated or sedated by alcohol were observed in participants following KS vs placebo. The lack of effects on subjective ratings of intoxication may be due to the low dose of alcohol that was administered (i.e., mean BrAC level of 0.02 in the placebo condition). Nevertheless, our findings suggest that KS modulates alcohol bioavailability and reduces the rewarding effects of alcohol (i.e., reduced alcohol liking and wanting). Because the KS used in the study contained high quantities of the sweetener allulose, which can be synthetically converted from allitol by alcohol dehydrogenase (Zhang et al., 2016), a key enzyme in alcohol metabolism, we also evaluated the potential confound that allulose alone may have on BALs in rats. We found that allulose had no effects on BAL in rats, whereas the KS decreased BAL, which further corroborated our findings that KS modulate alcohol bioavailability.

We postulate the following mechanisms by which KS reduces alcohol bioavailability. First, ketone D/L-3-hydroxybutyrate has been shown to delay gastric emptying in healthy humans (Rittig et al., 2020), and delayed gastric emptying is associated with lower alcohol absorption (Horowitz et al., 1989). Second, studies have shown that exogenous ketones increase levels of nicotinamide adenine dinucleotide (NAD+) (Elamin et al., 2017; Xin et al., 2018), a cofactor necessary for alcohol metabolism (Cederbaum, 2012), and increased NAD availability may accelerate alcohol metabolism in the liver (Wu et al., 2021). Third, 1,3-butanediol is catabolized to BHB by ADH (Tate et al., 1971) and may prime the activity of ADH for the subsequent metabolism of alcohol. In one study, in a subpopulation of individuals, prior exposure to alcohol increased the elimination rate of a second dose of alcohol by more than 40% above the basal rate (Thurman et al., 1989). Therefore, a potential prior activation of ADH, either by 1,3 butanediol, could increase the metabolic rate of alcohol. Further research is needed to elucidate the mechanisms underlying the effects of KS and to differentiate effects on alcohol absorption from those on metabolism.

Our finding that KS *reduced* BrAC and BAL in humans and rats contrasts with our previous findings that endogenous production of ketones following a 6-week ketogenic diet substantially increased BALs in rats exposed to alcohol vapor (Wiers et al., 2021). KS may slow down alcohol absorption rather than speeding up alcohol metabolism because alcohol administered through inhalation is absorbed mainly through the lungs, bypassing gastrointestinal absorption and first-pass metabolism in the stomach, and rapidly reaches the arterial circulation (Mouton et al., 2016; MacLean et al., 2017). Consistent with the present study results, oral administration of KS and alcohol in mice also led to significantly dampened BAL (Bornebusch et al., 2021). However, Bornebusch et al. also indicated that a 3-week ketogenic vs regular diet did not affectBAL following oral alcohol administration (Bornebusch et al., 2021). The difference in findings between the ketogenic diet and KS may also be due to the carbohydrate restriction required by the diet. Thus, it is possible that the carbohydrate restriction characteristic of the ketogenic diet, rather than the presence of ketone bodies per se, impaired alcohol metabolism in the rats. There is evidence that carbohydrates (Finnigan et al., 1998), including sucrose and fructose (Stamates et al., 2015; Smith et al., 2017), reduce BrAC and BAL due to both delayed absorption and increased availability of NAD+ for alcohol metabolism. Here, KS, without adherence to a carbohydrate-restrictive diet, elevated ketone levels. Moreover, studies have shown a greater ADH concentration in the liver of rats in the fed than the fasting state (Meijer et al., 1975; Cederbaum et al., 1977). Administration of a 6-week ketogenic diet could induce a fasting state resulting in increased ADH activity and a decreased rate of alcohol oxidation.

Previous clinical and preclinical studies have shown that a ketogenic diet decreases alcohol craving and alcohol consumption (Blanco-Gandía et al., 2021; Wiers et al., 2021). Although, herein, the KS reduced alcohol liking and wanting ratings even after controlling for t0 measures (i.e., 15 minutes after KS/placebo but before alcohol administration), we did not see significant effects of KS on alcohol craving scores as measured with the AUQ. The null finding is likely attributable to the inclusion of healthy individuals with low alcohol craving scores. Future studies are needed to examine the effects of KS on alcohol craving and alcohol consumption in individuals with AUD and in preclinical models of alcohol dependence. Interestingly, we did not find significant effects of the KS on subjective feelings of intoxication. The low dose of alcohol used in this study may not have been sufficient to elicit feelings of intoxication because the BAES responses during the alcohol challenge did not change over time. Furthermore, exploratory analyses did not show significant correlations between BrAC/BAL and subjective ratings of intoxication on the BAES or DEQ (supplementary Table 2). The presence of sucrose in our placebo drink may also have dampened the intoxicating effects of alcohol (Stamates et al., 2015; Smith et al., 2017) and contributed to the absence of differences between KS and placebo. Future studies should examine the effects of KS on both subjective and objective responses to a higher dose of alcohol.

Consistent with Stubbs et al. (2017), we demonstrated that a single dose of a KS was effective in elevating blood BHB levels and decreasing blood glucose within 30 minutes of consumption. Several incidences of hypoglycemia (glucose <70 mg/dL) were observed during the study that may be attributable to the KS alone or the interaction of KS with alcohol. Stubbs et al. (2017) found that KS increased insulin levels, which could account for the reduced glucose levels. In their seminal paper, Krebs et al. (1969) found that alcohol inhibits gluconeogenesis, which produces glucose from noncarbohydrate carbons (i.e., lipids and proteins), and these findings were later replicated in human studies that measured gluconeogenic flux following radiolabeled [2-^13^C_1_]glycerol infusion (Siler et al., 1998). Although suppression of gluconeogenesis may not be concerning under normal physiological conditions when glucose is readily available, the effect may be compounded by alcohol consumption combined with KS, which together may give rise to hypoglycemia. None of our participants reported symptoms of hypoglycemia (e.g., dizziness and shakiness), likely because the high levels of ketones provided an alternative to glucose as a source of energy. Nonetheless, the effect of KS on glucose levels raises potential health concerns, for example in individuals with metabolic disorders such as diabetes. Furthermore, KS-associated gastrointestinal discomfort was reported in our study and others (Stubbs et al., 2019), but the effects were mild and transitory and may be attributable to the ketones, allulose, or both. Although KS decreased blood glucose levels in humans, there were no effects of KS on blood glucose levels in rats, suggesting species differences in ketone metabolism. Previous rat studies demonstrated both null effects (Kesl et al., 2016) and reduced glucose levels in response to ketone supplementation (Ari et al., 2016; Kesl et al., 2016; Brownlow et al., 2017), which may be attributable to different KS used, chronic vs acute administration, and other variations in experimental procedures, such as dietary intake (e.g., time since the last meal and dietary composition).

Our study has several limitations and raises questions for future studies. First, our sample of healthy individuals was small (*n* = 10), and future larger studies that include individuals with AUD are warranted to compare the effects of KS on alcohol-related measures in healthy and AUD individuals. Furthermore, low alcohol craving scores in our study population of healthy individuals likely resulted in a floor effect that obscured any effects of KS on alcohol craving. Second, we chose a low-alcohol dose due to concerns about heightened intoxication in individuals following KS, as was observed in rats on a ketogenic diet (Wiers et al., 2021). The low dose of alcohol may have been insufficient to elicit intoxicating effects because exploratory analyses yielded no correlation between BrAC/BAL and BAES and DEQ scores (supplementary Table 2). This could account for the null effects of KS on subjective reports of intoxication. Third, our placebo and KS were not calorically matched, which could have contributed to the observed differences in alcohol sensitivity. However, our placebo contained sucrose, which has been shown to reduce alcohol sensitivity (Stamates et al., 2015; Smith et al., 2017). We found that KS dampened BrAC and BAL more than a sugar-containing placebo, which testifies to the robustness of the effects of KS. Fourth, the mechanism(s) underlying the effects of KS on alcohol absorption and metabolism remain to be elucidated. In our study, compared with placebo, KS reduced peak BrAC and BAL, but exploratory data analyses revealed no difference in elimination rates [t(7) = 1.4, *P* = .2] (supplementary Analyses of Alcohol Elimination Rate; supplementary Figure 4). This may reflect an effect of KS in reducing alcohol absorption rather than altering metabolism (Jones, 2019), but future studies are warranted to differentiate the two mechanisms. Fifth, the taste of KS was difficult to conceal; thus, most participants correctly identified the KS and placebo interventions. Although we controlled for baseline measures (t0, post-KS/placebo intervention and pre-alcohol administration), it is possible that the unpleasant taste and nauseating effects of KS could have confounded subjective responses to alcohol. Lastly, sex differences in alcohol metabolism have been previously documented, with higher blood alcohol concentration in women due to faster alcohol absorption and sex differences in body composition (Cederbaum, 2012). Although our rat experiment demonstrated a main effect of sex on BALs, our human experiments adjusted the alcohol dose for sex but were underpowered to test for sex differences on alcohol responses due to the low number of female participants. In rats, no sex × ketone treatment interaction was found, suggesting an effect of the ketone treatment irrespective to sex, but this requires further elucidation in humans.

In sum, we found that the administration of an exogenous KS attenuates BrAC and BALs following an alcohol challenge. Regarding the subjective responses to alcohol, KS did not alter ratings of stimulation or sedation on the BAES or FEEL and HIGH on the DEQ, but it increased DISLIKE and decreased LIKE and liking MORE on the DEQ. Binge alcohol drinking and AUD are prevalent in the United States (Substance Abuse and Mental Health Services Administration, 2022), and alcohol intoxication increases the risks for alcohol poisoning and reckless behaviors that are harmful to oneself or others (Esser et al., 2020). Therefore, KS may reduce alcohol intoxication and alcohol-related harm by reducing BALs.

## Supplementary Material

pyae009_suppl_Supplementary_Figures_S1-S5_Tables_S1-S2

## Data Availability

The data underlying this article will be shared on reasonable request to the corresponding author.

## CRediT

XL, TP, RPRN, HRK JCMV, LFV, and CEW contributed to the study conceptualization. XL, DT, TP, JB, SV, RR, GK, CEW, LFV and JCMV were responsible for the investigation. XL, ZS, GK, VR, HRK, JCMV, LFV, and CEW performed formal analysis. XL, ZS, DT, and CEW drafted the manuscript and all authors provided edits to the manuscript.
