# Supplementary material for: Ketone Supplementation Dampens Subjective and Objective Responses to Alcohol: Evidence From a Preclinical Rat Study and a Randomized, Cross-Over Trial in Healthy Volunteers
Source: Int J Neuropsychopharmacol. 2024 Feb 5;27(2):pyae009. doi: 10.1093/ijnp/pyae009 (PMC10901540; doi:10.1093/ijnp/pyae009)
Supplement: pyae009_suppl_Supplementary_Figures_S1-S5_Tables_S1-S2 [file pyae009_suppl_supplementary_figures_s1-s5_tables_s1-s2.docx]

**Ketone supplementation dampens subjective and objective responses to alcohol: evidence from a preclinical study and a randomized, cross-over trial in healthy volunteers**

Xinyi Li^1^, Zhenhao Shi^1^, Dustin Todaro^1^, Timothy Pond^1^, Juliana Byanyima^1^, Sianneh Vesslee^1^, Rishika Reddy^1^, Gabriel Kass^1^, Ravi Prakash Reddy Nanga^2^, Vijay Ramchandani^3^, Henry R. Kranzler^1^, Janaina Vendruscolo^4^, Leandro Vendruscolo^4^, Corinde E. Wiers^1^

^1^Center for Studies of Addiction, University of Pennsylvania Perelman School of Medicine, Department of Psychiatry, Philadelphia, PA

^2^University of Pennsylvania Perelman School of Medicine, Department of Radiology, Philadelphia, PA

^3^National Institute on Alcohol Abuse and Alcoholism, National Institutes of Health, Bethesda, MD

^4^National Institute on Drug Abuse, National Institutes of Health, Baltimore, MD

**SUPPLEMENTARY MATERIALS**


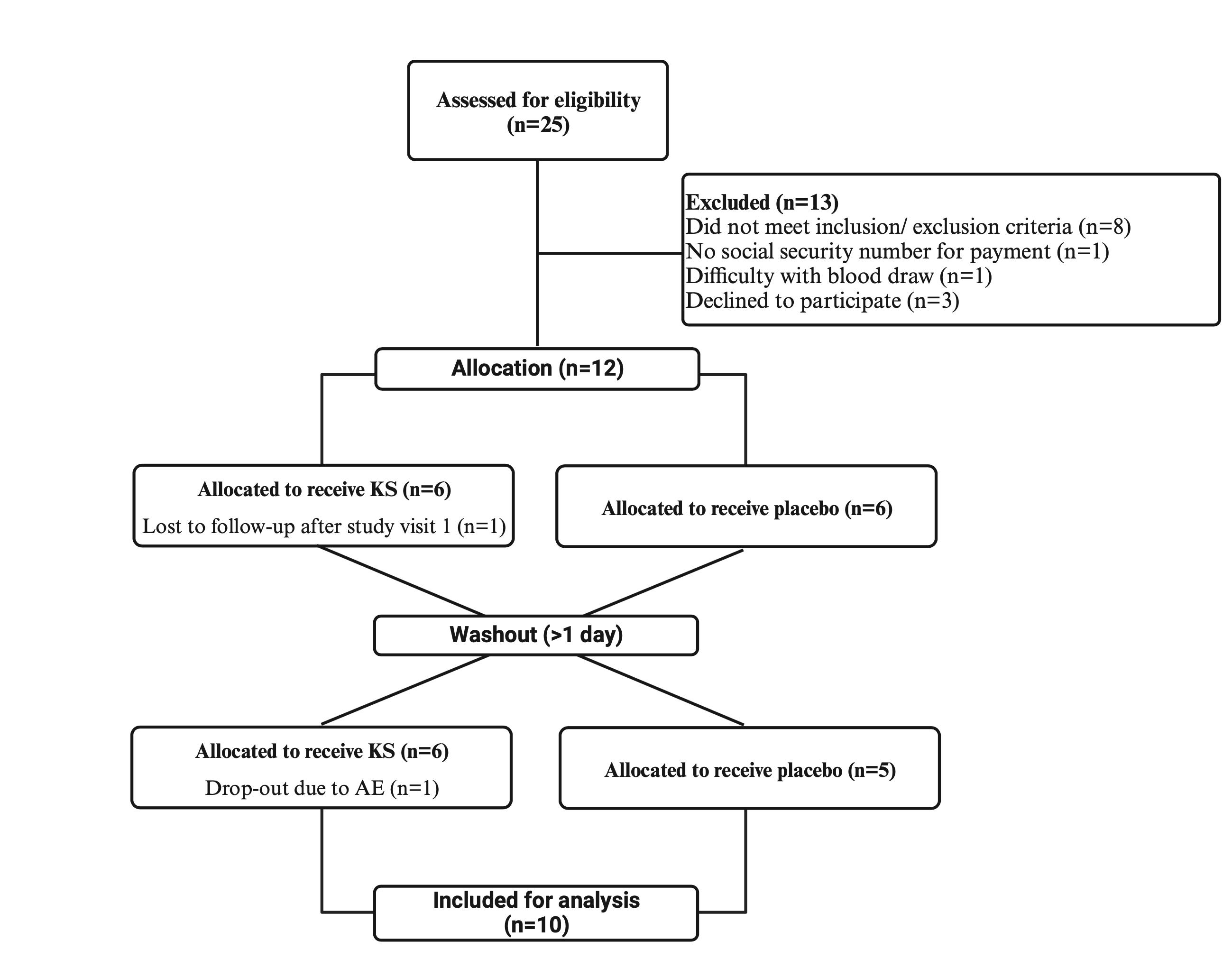


**Figure S1: CONSORT Diagram.** Participants were recruited in the greater Philadelphia, PA area. Of the 25 screened participants, 12 participants were allocated to receive KS and placebo and completed the first study visit. One participant was lost to follow-up and 11 participants returned for the second study visit. One participant vomited following ingesting the KS drink and dropped out of the study. A total of 10 participants who completed the study were included for analysis. Abbreviation: KS, ketone supplement.

**Table S1**. Summary of adverse events.

| **Number of participants** | **Event** | **Severity** | **Relatedness to KS** |
| --- | --- | --- | --- |
| 3 (including 1 drop out) | Gastrointestinal discomfort: nausea, stomachache, diarrhea, and vomiting (drop-out) | Mild | Probable |
| 1 | Fainting during blood draw | Mild | Unlikely |
| 5 | Glucose < 70 mg/ dl during at least 1 time point during the study | Mild | Probable |

**Figure S2**. **Individual breath alcohol concentration following an alcohol challenge in individuals administered KS or placebo.** BrAC was measured prior to (t0) and 15, 30, 45, 60, 90, 120, 150, and 180 min following alcohol administration. Abbreviations: KS, ketone supplement.

**Analyses of Baseline-Adjusted DEQ Scores**

For “FEEL(ING) the effects of alcohol”, there was significant effect of Time (F_3,59.3_= 5.7, *p* =0.002), but not Intervention (F_1,60.7_= 0.6, *p* =0.4) or Time x Intervention (F_3,59.3_= 0.4, *p* =0.7). Reports of “HIGH” throughout the alcohol challenge did not differ with effects of Intervention (F_1,63.4_= 1.0, *p* =0.3), Time (F_3,60.0_= 0.9, *p* =0.4), or Time x Intervention (F_3,60.1_= 0.3, *p* =0.8). For “DISLIK(ING) any of the effects you are feeling”, there was a significant effect of Intervention (F_1,58.8_=8.8, *p* =0.004), but no effects of Time (F_3,52.8_= 0.7, *p* =0.5) or Intervention x Time interaction (F_3,52.7_= 0.2, *p* =0.9). Reports of “LIK(ING) any of the effects you are feeling” demonstrated an effect of Intervention (F_1,53.4_=5.7, *p* =0.02), but no significant effects of Time (F_3,53.2_= 0.3, *p* =0.8) or Intervention x Time interaction (F_3,53.2_= 0.2, *p* =0.9) were observed. For responses to “lik(ing) MORE of what you consumed”, there was a significant effect of Time (F_3,57.9_= 3.0, *p* =0.039), but not Intervention (F_1,65.6_=0.0, *p* =0.9) or Intervention x Time interaction (F_3,57.9_= 0.1, *p* =1.0).

**Analyses of BAES and AUQ**

On the BAES, paired t-test did not reveal significant baseline (t0) differences on subjective reports of stimulation [“energized,” “excited,” and “up”, t(9)=1.2, *p* =0.2) or sedation [“sedated”, “slow thoughts”, and “sluggish”, t(9)=1.6, *p* =0.2). Reports of stimulation did not differ with effects of Intervention (F_1,61.2_=3.4, *p* =0.07), Time (F_3,61.1_=0.2 *p* =0.9) or Intervention x Time interaction (F_3,61.1_=31.0 p=0.4), even after adjusting for baseline responses [Intervention: (F_1,66.4_=0.001, *p* =0.98); Time: (F_3,60.8_=0.3, *p* =0.8); Intervention x Time interaction (F_3,60.8_=1.3 *p* =0.3)]. Reports of sedation did not significantly differ with effects of Intervention (F_1,61.1_= 1.6, p=0.2), time (F_3,61.0_= 0.5, p=0.7), or Intervention x Time interaction (F_3,61.0_= 0.4, p=0.8) and no significant effects were observed after adjusting for baseline responses [Intervention (F_1,64.3_= 1.4, *p* =0.2); Time (F_3,59.9_= 0.7, *p* =0.6); Intervention x Time interaction (F_3,59.9_= 0.6, *p* =0.6) (see **Figure S3A**).

Regarding participants’ responses on the AUQ, no significant effects of Intervention (F_1,44.0_= 0.2, *p* =0.7), Time (F_2,44.0_= 0.4, *p* =0.7), and Intervention x Time interaction (F_2,44.0_= 0.5, *p* =0.6) were observed (see **Figure S3B**).

**Figure S3**. **Subjective responses following an alcohol challenge in individuals administered KS and placebo.** (A) BAES responses before and 30, 60, 90, and 180 min following an alcohol challenge. (B) AUQ responses before and 90 and 180 min after an alcohol challenge. Mean ± SEM. Abbreviations: AUQ, alcohol urge questionnaire; BAES, brief biphasic alcohol effect scale; KS, ketone supplement.

**Table S2**. Correlation between breath and blood alcohol levels with subjective report of intoxication.

| **Questionnaire items** | **Breath alcohol (%)** | **Blood alcohol (mg/dL)** |
| --- | --- | --- |
| ***BAES*** |  |  |
| Stimulation | R²=0.1, *p* =0.2 | R²=0.04, *p* =0.3 |
| Sedation | R²=0.05, *p* =0.8 | R²=0.01, *p* =0.7 |
| ***DEQ*** |  |  |
| Do you FEEL the effects of alcohol right now? | R²=0.06, *p* =0.3 | R²=0.09, *p* =0.3 |
| Are you High, right now? | R²=0.00, *p* =1.0 | R²=0.01, *p* =0.7 |
| Do you DISLIKE any of the effects you are feeling? | R²=0.01, *p* =0.6 | R²=0.03, *p* =0.6 |
| Do you LIKE the effects you are feeling? | R²=0.01, *p* =0.6 | R²=0.06, *p* =0.4 |
| Would you like MORE of what you consumed right now? | R²=0.2, *p* =0.08 | R²=0.1, *p* =0.2 |

**Analyses of Alcohol Elimination Rate**

Alcohol elimination rates were calculated as per Jones, 2019. Individual Pearson correlation coefficients were extracted from linear regression between time and BrAC in the descending phase of the alcohol curve (i.e., following peak BrAC) and compared between KS and placebo intervention arms using paired t-test. We were unable to perform linear regression and calculate correlation coefficients for 2 participants during the KS intervention because they had BrAC of 0 throughout the alcohol challenge. We did not find a significant difference in elimination rate between the KS and placebo interventions (t_7_=1.4, *p* =0.2) (see **Figure S1**).

**Figure S4. Alcohol elimination rate following an alcohol challenge paradigm in individuals administered KS or placebo administration.** Abbreviations: KS, ketone supplement.

**Figure S5: Blood levels in rats administered KS, allulose, or water.** Measurements in males are displayed on the left and females on the right. T0 corresponds to the time of alcohol administration. **(A)** Blood glucose and **(B)** BHB levels were assessed immediately prior (t0, 30 min following KS, allulose, or water administration) and at 15-, 30-, 60-, 120-, and 240-min post-alcohol oral gavage administration. **(C)** Assessments for BAL were made at 15-, 30-, 60-, 120-, and 240- min after oral gavage administration of alcohol. Data are expressed as mean ± SEM. *Indicates sex differences at a particular time point, *p* <0.05. Abbreviation: BHB, β-hydroxybutyrate; KS, ketone supplement
